# Role of JNK isoforms in the development of neuropathic pain following sciatic nerve transection in the mouse

**DOI:** 10.1186/1744-8069-8-39

**Published:** 2012-05-22

**Authors:** Giusi Manassero, Ivan E Repetto, Stefano Cobianchi, Valeria Valsecchi, Christophe Bonny, Ferdinando Rossi, Alessandro Vercelli

**Affiliations:** 1 Department of Neuroscience, Neuroscience Institute of Turin (NIT), University of Turin, I-10125, Turin, Italy; 2 Neuroscience Institute of the Cavalieri-Ottolenghi Foundation (NICO), University of Turin, Regione Gonzole 10, 10043, Orbassano Turin, Italy; 3 Group of Neuroplasticity and Regeneration, Institute of Neuroscience-CIBERNED, Universitat Autonoma de Barcelona, E-08193, Bellaterra, Barcelona, Spain; 4 Division of Medical Genetics, CHUV-University Hospital, 1011, Lausanne, Switzerland

**Keywords:** Neuropathic pain, Sciatic nerve transection, JNK isoforms, Knockout mice

## Abstract

**Background:**

Current tools for analgesia are often only partially successful, thus investigations of new targets for pain therapy stimulate great interest. Consequent to peripheral nerve injury, c-Jun N-terminal kinase (JNK) activity in cells of the dorsal root ganglia (DRGs) and spinal cord is involved in triggering neuropathic pain. However, the relative contribution of distinct JNK isoforms is unclear. Using knockout mice for single isoforms, and blockade of JNK activity by a peptide inhibitor, we have used behavioral tests to analyze the contribution of JNK in the development of neuropathic pain after unilateral sciatic nerve transection. In addition, immunohistochemical labelling for the growth associated protein (GAP)-43 and Calcitonin Gene Related Peptide (CGRP) in DRGs was used to relate injury related compensatory growth to altered sensory function.

**Results:**

Peripheral nerve injury produced pain–related behavior on the ipsilateral hindpaw, accompanied by an increase in the percentage of GAP43-immunoreactive (IR) neurons and a decrease in the percentage of CGRP-IR neurons in the lumbar DRGs. The JNK inhibitor, D-JNKI-1, successfully modulated the effects of the sciatic nerve transection. The onset of neuropathic pain was not prevented by the deletion of a single JNK isoform, leading us to conclude that all JNK isoforms collectively contribute to maintain neuropathy. Autotomy behavior, typically induced by sciatic nerve axotomy, was absent in both the JNK1 and JNK3 knockout mice.

**Conclusions:**

JNK signaling plays an important role in regulating pain threshold: the inhibition of all of the JNK isoforms prevents the onset of neuropathic pain, while the deletion of a single splice JNK isoform mitigates established sensory abnormalities. JNK inactivation also has an effect on axonal sprouting following peripheral nerve injury.

## Background

Neuropathic pain refers to a variety of chronic pain conditions that are mediated via different pathophysiological mechanisms [1]. Our incomplete understanding of its pathogenesis has hampered the development of effective treatments for this terribly disabling disease. Since sensory neurons connect peripheral tissues with the central nervous system, they play a key role in the reception of peripheral information, and in its rapid transduction and integration [2]. Transection of peripheral axons of primary sensory neurons, an established model for the study of neuropathic pain, results in profound alterations in sensory neuron metabolism, in their survival and regenerative potential, excitability, transmitter function, and in their sensitivity to diverse extrinsic and intrinsic signals [3]. When axons are sectioned, sensory neurons undergo long- lasting plastic changes in their neurochemical and electrical properties [4], which can result in neuropathic pain, placing a disabling burden on the affected individual [2,5].

Damage to peripheral nerves leads to a rapid influx of Ca++ and Na+, which elicits electrical responses that back-propagate to the cell body in the dorsal root ganglion (DRG) and contribute to the activation of several protein kinase pathways, including protein kinase (PK) A, PKC, and mitogen-activated protein kinases (MAPKs) [5,6].The MAPK family comprises three major members: extracellular signal-regulated kinase (ERK), p38, and c-Jun N-terminal kinase (JNK), all of which are implicated in the nociceptor sensitization that is associated with inflammation and peripheral neuropathy [7-9].

An increasing body of evidence suggests that the JNK cascade is a critical signaling pathway for the onset and the maintenance of neuropathic pain, via distinct mechanisms in the cells of the DRGs and spinal cord: following nerve injury, JNK is rapidly activated by phosphorylation, and JNK expression remains elevated for weeks, until the neuron dies or until its axon regenerates [9-11]. Recently, a cell- penetrating and proteinase-resistant peptide inhibitor of the JNK binding domain of JNK-interacting protein-1 (D-JNKI-1) was developed, with the goal of competitively inhibiting the binding of JNK to its substrates [12-14]. D-JNKI-1 effectively blocks the onset of mechanical allodynia, a characteristic behavioral response for neuropathic pain [9,15]. However, D-JNKI-1 treatment did not permanently prevent neuropathic pain: allodynia re-appeared when the drug was discontinued [9].

The JNK protein kinase in mammals is encoded via three genes: *Jnk 1* and *Jnk 2* are ubiquitously expressed, while *Jnk 3* is expressed primarily in the nervous system, endocrine pancreas, heart, and testicles [16,17]. The JNK isoforms differ from each other in the affinity of their binding with the Activating Transcription Factor (ATF) 2, with EtsLiKe gene (Elk)-1, and with c-Jun transcription factors. Individual JNKs may thus selectively target specific transcription factors *in vivo*[18]. However, the relative contribution of each JNK isoform to the onset and maintenance of neuropathic pain remains to be identified.

In this study, we use unilateral sciatic nerve transection (SNT) in knockout (KO) mouse models of neuropathic pain, in combination with JNK inhibition with D-JNKI-1, to analyze the contribution of individual JNK isoforms to the animal’s physiology and behavior. We show that following SNT, activated JNK induces the up-regulation and phosphorylation of the transcription factor c-Jun in the nucleus of DRG neurons, leading to the formation of an Activator Protein (AP)-1 complex that induces a number of downstream genes [10,19]. We further show that this neuronal response to injury comprises a set of changes that are primarily aimed at restoring disrupted connections and recovering function. In spite of the clear, adaptive nature of this response, some of its outcomes, such as abnormal sensitivity to peripheral stimuli [20], are clearly maladaptive; this led us to examine also the expression of growth associated protein (GAP)-43 and Calcitonin Gene Related Peptide (CGRP) in the DRGs, as indicators of injury-induced molecular changes [20,21] that could be related to the alterations in sensory function. Indeed, a relationship among the JNK pathway and GAP-43 [10,22] and CGRP [23] expression has been reported.

## Results

### Basal expression of JNK isoforms in the adult mouse DRGs

**Figure 1:**
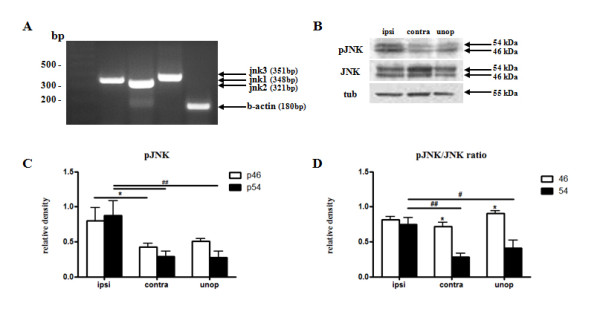
**Basal expression of JNK in the adult mouse DRGs.** (**A**) m-RNA expression of Jnk1, Jnk2 and Jnk3 in lumbar mouse DRGs, analyzed by RT-PCR. β-actin mRNA expression was used as the internal standard. (**B**) Western blot analysis of pJNK and JNK in the L3-L6 DRGs from SNT (ipsilateral and contralateral) and unoperated wt mice. (**C-D**) Densitometric analysis of pJNK (**C**) and pJNK/JNK ratio (**D**) p46 (white bars) and p54 (black bars) isoforms in SNT (ipsi: ipsilateral; contra: contralateral) and unoperated (unop) wt mice. Experiments were repeated three times. Data represent means + SEM (n = 24 from three independent experiments). *, # p < 0.05; ## p < 0.01 by one-way ANOVA.

Several studies have reported on JNK pathway activation in DRG neurons [9,10,22], but data on the basal mRNA expression of JNK isoforms in adult mouse DRGs are lacking. We first evaluated this basal expression in adult wild-type (wt) mice by qualitative RT-PCR (Figure 1A), then used western blotting to determine basal expression of phosphorylated (p) and total JNK proteins in adult wt DRGs: pJNK was present as a stronger (46 kDa) and a weaker (54 kDa) isoform, whilst total JNK was present as a weaker (46 kDa) and a stronger (54 kDa) isoform (Figure 1B). pJNK increases after STN (p46 0.80 + 0.11, p < 0.05; p54 0.88+ 0.12, p < 0.01, one-way ANOVA vs wt contralateral side) (Figure 1B-C). The increase in the pJNK/JNK ratio is mostly due to changes in p54 isoform (p54/54 0.75 + 0.10, one-way ANOVA, p < 0.01 vs wt contralateral side; p < 0.05 vs wt unoperated mice) (Figure 1D).

### D-JNKI-1 treatment

Preliminary experiments on intact wt animals that were systemically treated with the JNK peptide inhibitor D-JNKI-1, or with vehicle (saline), did not show any significant difference in GAP43 and CGRP-immunoreactivity (IR) in DRGs, nor did they show alteration in pain sensitivity between the two groups of mice (data not shown). Thus the D-JNKI-1 treated mice and the KO groups were compared to the same group of wt mice group, in order to minimize the number of animals used.

### Increase of GAP43-IR in wt DRG neurons is prevented by JNK blockade

**Figure 2:**
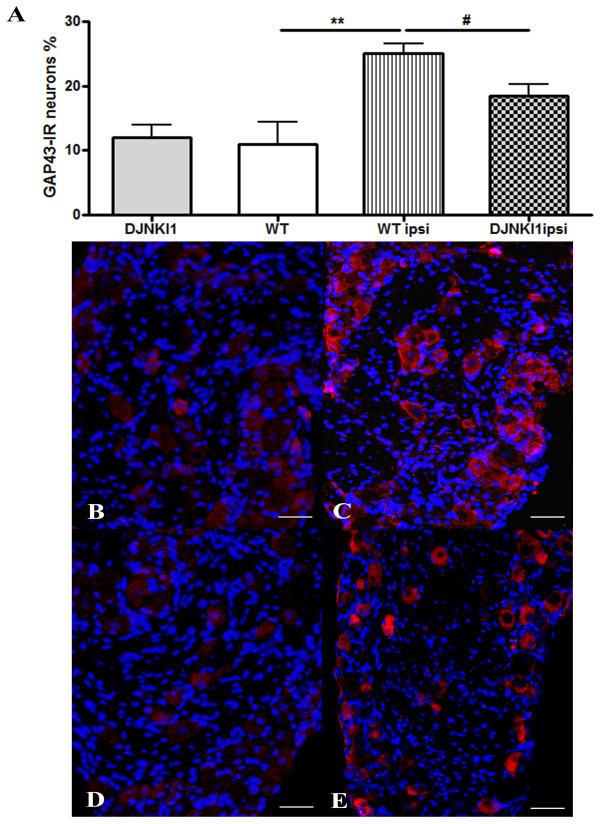
**GAP43-IR downregulation is partially prevented by JNK blockade in L4 primary sensory neurons of wt mice.** (**A**) The effect of D-JNKI-1 on SNL‒induced GAP43 expression in L4 DRG neurons. The immunoreactivity for GAP43 in DRG neurons was quantitated with the Neurolucida software, and expressed as a percentage of positive neurons. **p < 0.01 by tTest compared with unoperated wt; n = 8. #p < 0.05 by tTest compared with SNL ipsi; n = 8. Values are mean + SEM. (**B-E**)Representative DRG sections immune-stained for GAP43: (**B**) unoperated wt displays faint immunohistochemical labelling; (**C**) a significant increase in GAP43-IR occurred in DRGs, 72h after nerve injury; (**D**) D-JNKI-1 alone has no effect on baseline GAP43 levels; (**E**) D-JNKI-1 treatment partially prevents GAP43-IR upregulation on the lesioned side. Scale bar = 50 μm.

**Figure 3:**
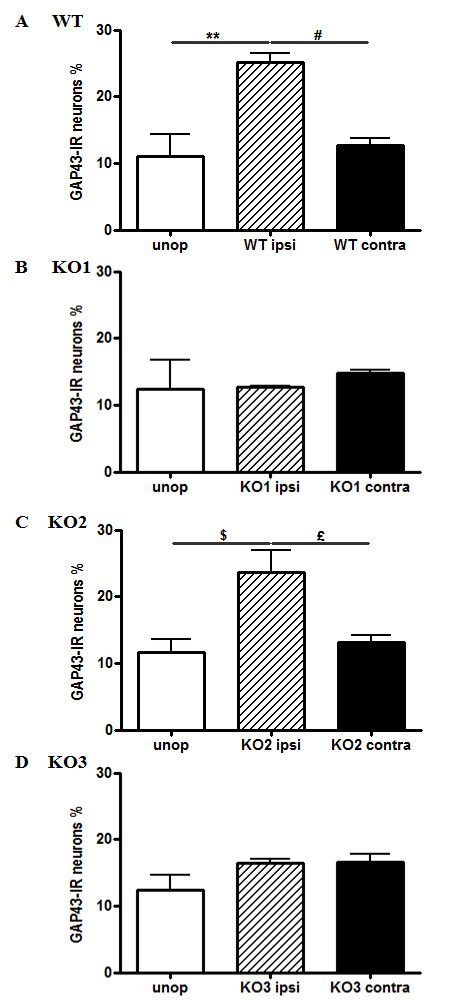
**GAP43-IR in L4 primary sensory neurons of JNK KO mice following STN.** Percentage of GAP-43-positive primary sensory neurons in L4 DRG in wilde type (**A**), JNK1 (**B**), JNK2 (**C**) and JNK3 (**D**) KO mice. GAP43 is constitutively expressed in primary sensory neurons of unoperated mice of each group (white bars). Seventy-two hours post lesion, GAP43-IR upregulation, which occurs in wt (**A**) and JNK2 KO (**C**) mice is absent both in JNK1 (**B**) and JNK3 (**D**) KO mice. #, £, $ p < 0.05, **p < 0.01 by tTest; n = 6 mice/group.

GAP43 is an axonal protein that is developmentally regulated, and is reactivated in some adult neurons following injury [25,26]. We evaluated GAP43 expression in L4 DRGs 72h post-surgery. In unoperated mice, an average of 11.03% + 3.37 neurons in L4 DRGs of both sides display some faint immunohistochemical labelling (Figure 2A2B). At 72h, GAP43-IR was increased ipsilateral to the SNT, compared to the unoperated group (25.11% + 1.40, t-test, p < 0.01) (Figure 2A2C): the contralateral DRG showed no change (12.73% + 1.07) (Figure 3).

Since peripheral nerve injury induces JNK and c-Jun activation in DRG neurons [9], and since it has been proposed that c-Jun regulates the expression of growth-associated genes [10,22], we asked whether blockade of the JNK pathway affects injury-induced GAP43 activation. D-JNKI-1 treatment significantly reduced the number of GAP43- immunoreactive neurons (18.54% + 1.66 on the operated side, t-test, p < 0.05 vs wt) (Figure 2A2E), whereas D-JNKI-1 had no effect on baseline levels of GAP43 (11.3% + 2.71 on the control side, p = 0.82 vs unoperated mice) (Figure 2A2D).

### GAP43-IR in DRG neurons of specific JNK KO mice following STN

The number of GAP43-IR neurons in L4 DRGs of unoperated mice that had the genes for the different JNK isoforms deleted was similar to that of unoperated wt animals (JNK1 KO 12.38% + 4.32; JNK2 KO 11.61% + 1.97; JNK3 KO 11.24% + 2.75, one-way ANOVA, p = 0.99) (Figure 3A). At 72h post-transection, GAP43-IR was unchanged in both JNK1 and JNK3 KO mice (JNK1 KO: 12.66% + 0.26, p = 0.57 vs JNK1 KO unoperated mice; JNK3 KO: 18.83% + 1.95, p = 0.28 vs JNK3 KO unoperated mice) (Figure 3B, 3D). On contrast, immunoreactivity in JNK2 KO mice was similar to that of the operated wt animals (23.69% + 3.21, t-test p < 0.05 vs JNK2 KO unoperated mice) (Figure 3C).

### CGRP-IR downregulation in wt DRG neurons is prevented by JNK blockade

**Figure 4:**
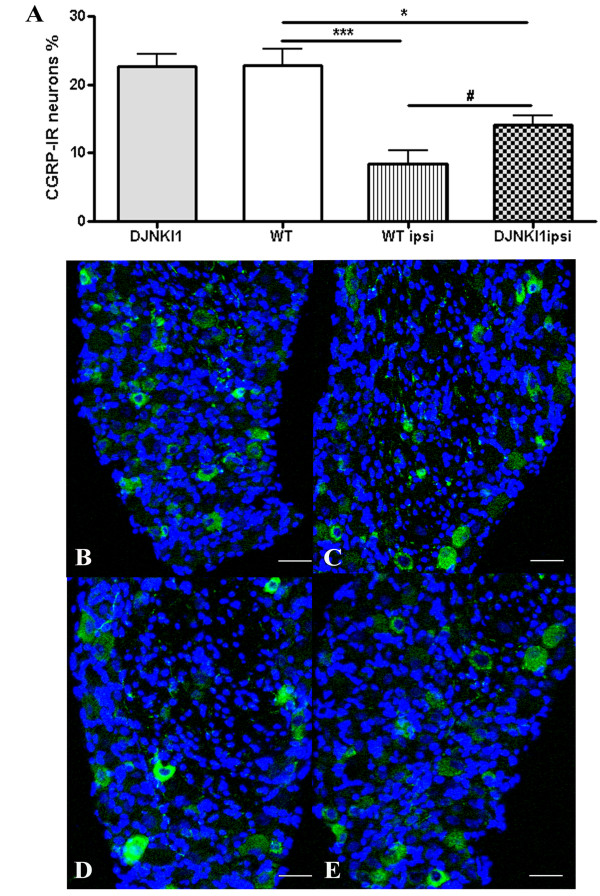
**CGRP-IR downregulation is prevented by JNK blockade in L4 primary sensory neurons of wt mice.** (**A**) Effect of D-JNKI-1 on SNT‒induced CGRP expression in L4 DRG neurons. CGRP-IR in neurons was quantitated with the Neurolucida software, and expressed as a percentage of positive neurons. *p < 0.05 and ***p < 0.001 by tTest compared with unoperated wt; n = 11. #p < 0.05 by tTest compared with SNL ipsi; n = 8. Values are mean + SEM (**B-E**). Representative 20X DRG sections immunoistochemically stained for CGRP: (**B**) baseline expression of CGRP-IR in the DRGs of the wt mice; (**C**) a significant decrease in CGRP-IR occurs in injured DRGs after 72h; (**D**) D-JNKI-1 alone has no effect on baseline CGRP-IR levels; (**E**) D-JNKI-1 treatment prevents downregulation of CGRP-IR. Scale bar = 50 μm.

CGRP is constitutively expressed in L4 primary sensory neurons (22.83% + 2.45) (Figure 4A4B), and is mainly localized in small- and medium-sized cells, as well as in some large neurons. Scattered sensory fibers in these DRGs are also immunolabeled. Since axotomy induces a time-dependent decrease in CGRP-IR in primary sensory neurons [23], we assessed CGRP-IR 72h post-lesion. CGRP-IR was decreased ipsilateral to the SNT, compared to the unoperated mice and also compared to the contralateral side (8.37% + 1.11, t-test, p < 0.001 vs unoperated mice) (Figure 4A4C). Pretreatment with D-JNKI-1 partially reversed the CGRP downregulation (14.10% + 1.31, t-test, p < 0.05, vs wt ipsilateral side) (Figure 4A4E). D-JNKI-1 alone had no effect on baseline levels of CGRP (22.65% + 1.89, t-test, p = 0.96 vs unoperated mice) (Figure 4A4D).

### CGRP-IR in DRGs of JNK KO mice following STN

**Figure 5:**
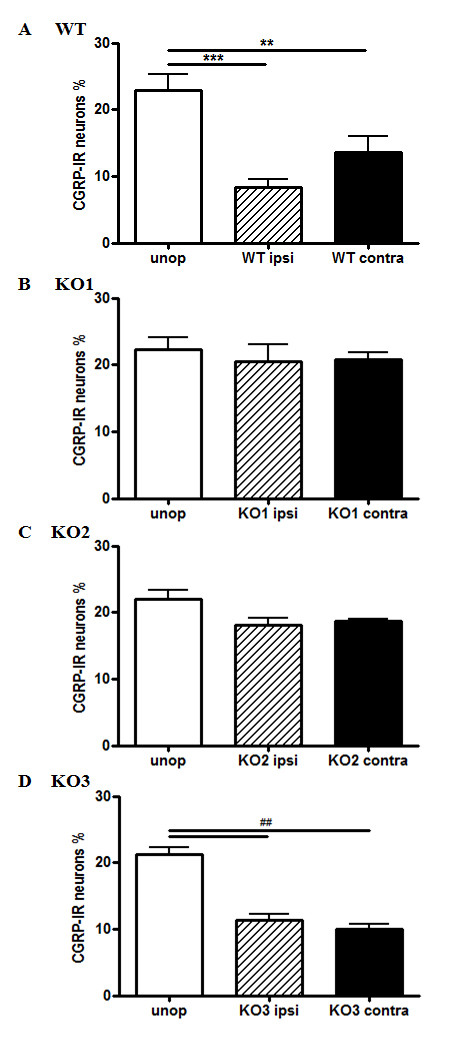
**CGRP-IR following injury in L4 primary sensory neurons of JNK KO Mice.** Percentage of CGRP-positive primary sensory neurons in L4 DRG in wt (**A**), JNK1 (**B**), JNK2 (**C**) and JNK3 (**D**) KO mice. CGRP is constitutively expressed in primary sensory neurons of unoperated mice of each group (white bars). Seventy-two hours post lesion, CGRP-IR , which is downregulated in wt (A) and JNK3 KO (**D**) mice, is unchanged both in JNK1 and JNK2 KO mice (**B, C**). ** and ##, p < 0.01 and ***p < 0.001 by tTest; n = 6 mice/group.

In JNK KO mice, the number of CGRP-IR neurons in L4 DRGs was unchanged relative to the intact wt DRGs (JNK1 KO 22.27% + 1.87; JNK2 KO 22.06% + 1.21; JNK3 KO 21.29% + 1.03, one-way ANOVA, p = 0.94) (Figure 5A). Seventy-two hours post-nerve transection, the frequency of CGRP-IR neurons was markedly decreased in JNK3 KO mice (11.31% + 1.54, t-test, p < 0.001 vs JNK3 KO unoperated mice), but not in JNK1 or JNK2 KO mice (JNK1 KO: 20.54% + 2.45, t-test, p = 0.81 vs JNK1 KO unoperated mice; JNK2 KO: 18.17% + 1.02, t-test, p = 0.12 vs JNK2 KO unoperated mice) (Figure 5B-D).

### Locomotor activity of unoperated JNK KO mice

To assess pain in terms of motor withdrawal in an animal model of injury, we first showed that motor functions were intact under basal conditions. Locomotor activity of mutant and wt mice was tested, by video and ink walking track analysis ( Additional file 1), by rotarod test and by open field test. Before injury, the toe spread and the print length average values of both sides in all mice, were approximately 0.65 cm and 1.45 cm, respectively, whereas the average stride length was 3.52 cm. In an open field box test, these parameters in JNK KO mice were undistinguishable from those of wt mice (p = 0.91, one-way ANOVA) ( Additional file 2). Moreover, all mice were able to remain on the rotating rod for 300 seconds (n = 12 mice/group). KO littermates for the different JNK isoforms were undistinguishable from their wt counterparts in the normal cage environment.

### Response to mechanical stimulation is decreased in JNK KO mice following SNT

**Figure 6:**
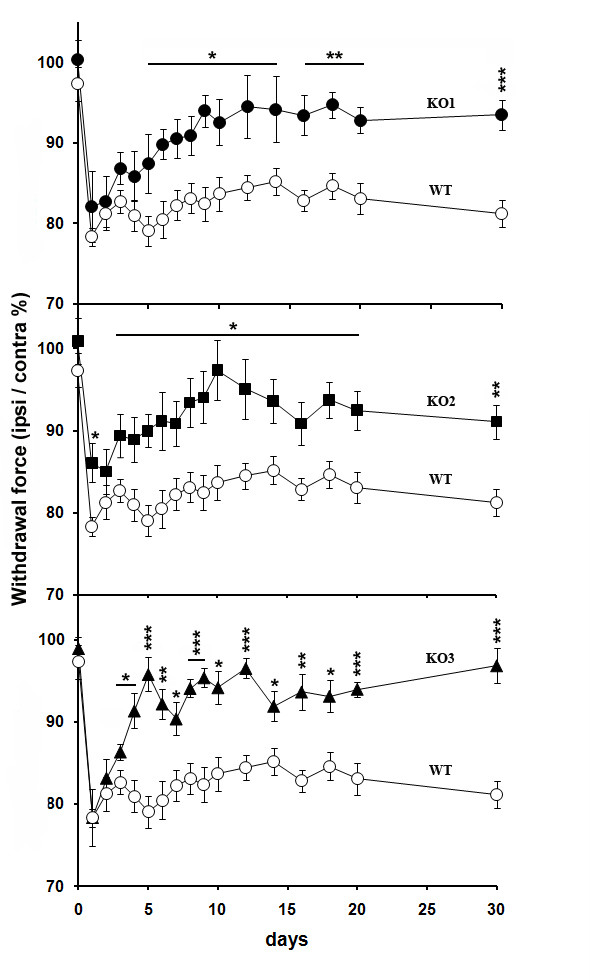
**Neuropathic pain behaviour in JNK KO and wt mice.** Mechanical hyperalgesia induced by SNT injury in JNK KO compared to wt mice: percentage ratio between ipsilateral and contralateral withdrawal thresholds in JNK1 KO (●), JNK2 KO (■), JNK3 KO (▴) and wt (○) mice. Asterisks indicate significant differences in percentage ratio of JNK KO vs wt mice (*p < 0.05; **p < 0.01; ***p < 0.001). n = 8 JNK1 KO, 12 JNK2 KO, 12 JNK3 KO, 13 wt.

The mechanical nociceptive threshold of both hindpaws, in response to dynamic plantar aesthesiometer stimulation, was measured in age-matched JNK KO and wt mice from 2 days prior to-SNT to 30 days post-SNT (Figure 6). Before nerve injury, paw withdrawal threshold to mechanical stimulation was comparable in all mice, suggesting that sensory transmission is not altered in the JNK KO mice. The time-course of the paw withdrawal threshold showed that, within 24 hours after cutting the sciatic nerve, the mechanical nociceptive threshold was decreased by 30-40% in all mice ipsilateral to the lesion, compared to the contralateral side; the foot withdrawal force was <7 g. We observed a mechanical hyperalgesia in the denervated hindpaw of the wt mice: this alteration persisted throughout the 30-day observation period (Figure 6, open symbols). By 72h, the paw withdrawal threshold in JNK1, JNK2 and JNK3 KO mice progressively increased ipsilateral to SNT (Figure 6A-C). Two-way ANOVA of repeated measures for mechanical hyperalgesia of ipsilateral hindpaws showed a significant main effect for groups, i.e. all individual JNK isoforms mutants vs wt (JNK1 KO: *F*_1,19_ = 17.444, p = 0.0005; JNK2 KO: *F*_1,23_ = 17.132, p = 0.0004; JNK3 KO: *F*_1,23_ = 39.203, p < 0.0001) and time (JNK1 KO: *F*_15,285_ = 8.127, p < 0.0001; JNK2 KO: *F*_15,345_ = 10.701, p < 0.0001; JNK3 KO: *F*_15,345_ = 13.938, p < 0.0001), in absence of significant differences for withdrawal force of contralateral hindpaws (JNK1 KO: *F*_1,19_ = 0.273, p = 0.6076; JNK2 KO: *F*_1,23_ = 2.019; p = 0.1688, JNK3 KO: *F*_1,23_ = 0.001; p = 0.9819) throughout the 30-day observation period. *Post-hoc* comparisons revealed significant differences among JNK2 KO vs wt mice from 24h to day 30, whereas JNK1 and JNK3 KO mice were significantly different from wt control from day 5 to day 30 (p < 0.05, Tukey-Kramer). We performed an additional statistical analysis comparing the withdrawal force of the ipsilateral with the contralateral hindpaw of JNK KO vs wt mice. Two-way ANOVA for repeated measures, followed by *post-hoc* comparisons, showed significant main effect for groups (JNK1 KO: *F*_1,19_ = 29.516, p < 0.0001; JNK2 KO: *F*_1,23_ = 18.532, p = 0.0003; JNK3 KO: *F*_1,23_ = 73.274, p < 0.0001) and time (JNK1 KO: *F*_15,285_ = 3.871, p < 0.0001; JNK2 KO: *F*_15,345_ = 3.439, p < 0.0001; JNK3 KO: *F*_15,345_ = 6.105, p < 0.0001) throughout the 30-day observation period (p < 0.05, Tukey-Kramer).

### Response to mechanical stimulation in wt mice after JNK blockade

**Figure 7:**
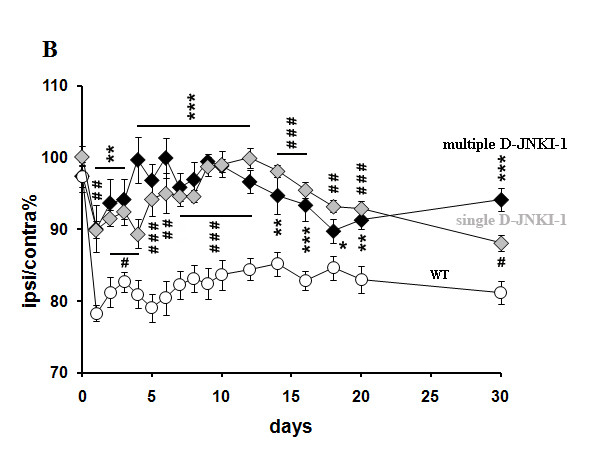
**Neuropathic pain behavior in wt mice after JNK blockade.** Mechanical hyperalgesia induced by SNT in wt mice is prevented by D-JNKI-1 administration. Percentage ratio between ipsilateral and contralateral withdrawal thresholds in wt mice injected with D-JNKI-1, after single (♦), or multiple (♦) injections, compared to wt (○). n = 12 single and n = 12 multiple injections, n = 13 wt.

To compare the behavioral effects of knocking out single JNK isoforms vs those resulting from JNK activity blockade, D-JNKI-1 was injected 30 min before SNT into a separate group of wt mice. Previous reports used different protocols of drug administration, such as systemic or intrathecal injection, to test the effect of D-JNKI-1 pain sensitization [9,15,27]. Here, we administered D-JNKI-1 sistemically before surgery to mimic the phenotype of a triple JNK1/2/3 KO mouse. In a first set of experiments (Figure 7, closed black symbols), we made repeated intraperitoneal injections of D-JNKI-1 every fourth day, starting from 30 min before surgery. The same delivery of D-JNKI-1 to unoperated animals did not alter basal pain sensitivity (data not shown). In mice that underwent SNT, two-way ANOVA of repeated measures for mechanical hyperalgesia of ipsilateral hindpaws yielded a significant main effect for multiple D-JNKI-1 injections (*F*_1,23_ = 52.361, p < 0.0001), time (*F*_15,345_ = 6.353, p < 0.0001) and interaction (*F*_15,345_ = 3.203, p < 0.0001), in absence of significant differences for withdrawal force of contralateral hindpaws (*F*_1,23_ = 0.296, p = 0.5914) throughout the 30-day observation period. *Post-hoc* comparisons showed that multiple D-JNKI-1 injections attenuated the onset of mechanical hyperalgesia at 24 h (p < 0.05, Tukey-Kramer). The anti-hyperalgesic effect of D-JNKI-1 reached a peak on day 5(p < 0.05, Tukey-Kramer), and remained elevated until day 12 (p < 0.05, Tukey-Kramer). This beneficial effect was still detectable,albeit it was reduced, at day 30 post-SNT (p < 0.05, Tukey-Kramer). Comparing the withdrawal force of the ipsilateral versus contralateral hindpaw of mice having received multiply D-JNKI-1 injects vs wt untreated mice, two-way ANOVA of repeated measures, followed by *post-hoc* comparisons, uncovered a significant main effect for treatment (*F*_1,23_ = 88.499, p < 0.0001), during the overall time course (p < 0.05, Tukey-Kramer).

Multiple D-JNKI-1 injections seem to have a cumulative effect on neuropathic pain symptoms [15]: neuropathic pain develops once peptide infusion is terminated [9]. We thus performed a second set of experiments, in which mice received only one injection of D-JNKI-1 30 min before surgery (Figure 7, closed grey symbols). Throughout the 30 days of observation, the latter protocol produced an anti- nociceptive effect on mechanical hyperalgesia similar to that observed following repeat peptide injections. Accordingly, the single-injection procedures induced a significant analgesic effect compared to wt untreated mice, as shown by *post-hoc* comparisons (p < 0.05, Tukey-Kramer). The time-course of the anti-hyperalgesic effect of the second treatment protocol was less fluctuating than the trend of the multiple D- JNKI-1 administration, and it was significantly different from the data obtained from SNT wt mice throughout the 30-day observation period (p < 0.05, Tukey-Kramer). Two-way ANOVA of repeated measures for mechanical hyperalgesia of ipsilateral hindpaws showed significant main effect for single D-JNKI-1 administration (*F*_1,23_ = 52.361, p < 0.0001) time (*F*_15,345_ = 6.353, p < 0.0001) and interaction (*F*_15,345_ = 3.203, p < 0.0001) during the overall time course. In the second treatment protocol, comparing the withdrawal force of the ipsilateral with the contralateral hindpaw of injected mice vs wt untreated mice, two-way ANOVA of repeated measures, followed by *post-hoc* comparisons, showed a significant main effect for treatment (*F*_1,23_ = 122.006, p < 0.0001) and time (*F*_15,345_ = 4.836, p < 0.0001) during the whole time course (p < 0.05, Tukey-Kramer).

### Autotomy behavior

**Figure 8:**
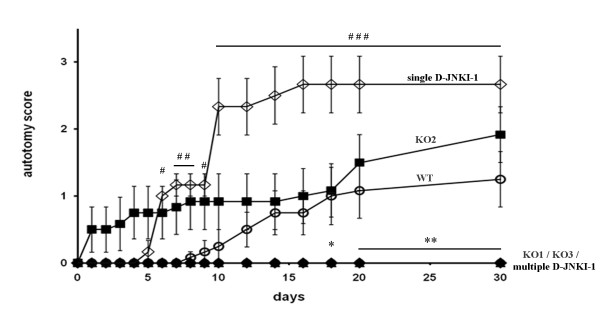
**Autotomy score.** Autotomy was scored throughout the 30-day observation period. Autotomy is highest in wt mice injected with D-JNKI-1 once (◊), next highest in JNK2 KO mice (■) and then in wt mice (○). Whilst regard to the time-course for onset of autotomy, JNK2 KOs (■) started to hurt their own ipsilateral hindpaws before wt injected with D-JNKI-1 once (◊) and in wt(○). This behavior was absent in both JNK1 (●) and JNK3 KO (▴), and in animals that received multiple D-JNKI-1 injections(♦). Autotomy scores are presented as mean + SEM.Significance:JNK1 KO, JNK3 KO and multiple D-JNKI-1 vs wt *p < 0.05; **p < 0.01; single D-JNKI-1 vs wt # p < 0.05; ## p < 0.01; ### p < 0.001, two-way ANOVA of repeated measures. n = same as in Figures 6 and 7.

The degree of self-mutilation, i.e. autotomy, of a denervated limb following axotomy is positively correlated to spontaneous pain [28]; this behavior has been observed even in humans suffering from neuropathic pain [29]. The time-course for the development of autotomy in all tested animals throughout the 30-day observation period is shown in Figure 8. Autotomy was most frequent in wt mice treated with a single D-JNKI-1 injection, followed by JNK2 KO mice and untreated wt mice. In fact, four days after administration, D-JNKI-1 is not active any more (our laboratory, unpublished observations). Some JNK2 KO mice started to hurt their own ipsilateral hindpaws the day after surgery, whereas in wt mice treated with a single D-JNKI-1 injection and in untreated wt mice, the first bites appeared at day 5 and day 8, respectively. Surprisingly, autotomy behavior was completely absent in both JNK1 and JNK3 KO mice, and in the animals had received multiple D-JNKI-1 injections.

## Discussion

Our overarching hypothesis is that the signals resulting in nociceptor sensitization are not separated, since the PKA, PKC, and MAPK cascades, once activated, partially or completely converge to induce hyperalgesia [2]. The JNK pathway is believed to be a critical signaling pathway in the processing of neuropathic pain via distinct mechanisms in the DRGs and spinal cord [9,10,24]. In this study, we used single JNK knockout mice to analyze the contribution of individual JNK isoforms to the development of neuropathic pain after unilateral SNT. We also studied the relationship between JNK activity and the expression of GAP-43 and CGRP, in order to relate changes at the molecular and cellular levels to the development of sensory alterations.

Our findings document, for the first time, that: (1) JNK is involved in pain–related behavior and in the modulation of GAP43 and CGRP expression in lumbar DRG neurons following SNT; (2) administration of a JNK inhibitor partially prevents these effects; (3) the deletion of a single specific JNK isoform partially prevents the maintenance of the sensory alteration, but not the onset of neuropathic pain symptoms such as mechanical hyperalgesia; (4) JNK inhibition in wt mice, as well as deletion of JNK1 or JNK3, abolishes SNT-induced autotomy behavior.

### The experimental model

Animal models of neuropathic pain are mostly based on peripheral nerve injury for reproducibility and simplicity [30]. Axotomy was the first widely used model, since it simulated the clinical condition of amputation [31]. Complete nerve transection is associated with spontaneous pain-related behaviors, allodynia and hyperalgesia, and with autotomy, or the self-mutilation of the injured foot. These three behavioral signs are used as indices to measure the degree of neuropathic pain, and to evaluate the effects of therapy [30]. In 2008, the International Association for the Study of Pain (IASP) modified many of the definitions in the pain field including that: i) the term allodynia should be used only when the test stimulus, e.g. thermal or mechanical, does not normally activate nociceptors; ii) when this is not clearly the case, “hyperalgesia” is the preferred term [32]. For this reason, in this study we analyzed the onset and maintenance of neuropathic pain in terms of mechanical hyperalgesia, since we tested the animal’s behavior by means of anautomated Von Frey test, which uses mechanical stimulation.

### Modulation by JNK of the molecular mechanisms involved in neuropathic pain

In this study we have focused our attention on the primary sensory neurons of the lumbar tract, which are the first neurons involved in pain transmission from the hind limb periphery. Following peripheral axotomy, reorganization of central DRG projections to the spinal cord are thought to mediate neuropathic pain [4]. Earlier studies proposed different explanations of how neuronal perikarya can receive information about a distant axon injury, including via retrograde transport of injury-induced proteins and/or via decreased target-derived trophic support [26,33].

It has been shown, both *in vitro* and *in vivo,* that axotomy of sensory neurons causes a rapid, massive and transient increase in JNK expression; this is followed by the activation of c-Jun, which supports axonal outgrowth and neuron survival [34,35]. In fact, JNK blockade by D-JNKI-1 and SP600125 reduces c-Jun phosphorylation in DRGs, and hampers axonal outgrowth, both in terms of the length and number of regenerating axons [35].

Many JNK scaffold proteins interact with kynesin 1, which is involved in vesicular trafficking within axons [36]. JNK is activated locally in the axon as part of a damage signal, which is retrogradely transported in a molecular assembly called a signalosome [37]. In cultured cortical neurons, JNK-interacting protein (JIP)1 has been shown to be involved in axonal elongation, since it is localized at the tip of the growing axon, and co-labels with the axonal marker Tau-1 [38]. Chang et al. [39] demonstrated that JNK1 KO mice exhibit progressive degeneration of neurites, associated with shorter microtubules,reduced Microtubules Associated Protein (MAP)1B, and MAP2 phosphorylation. MAP1-B is implicated in axonal regeneration, as seen in DRG cultures [40] and its phosphorylation is increased in the rearrangement of axonal circuitry following spinal cord injury, together with JNK activation [41]. All three JNK isoforms seem to contribute to neurite re-growth in PC12 cell culture [42]. The absence of the JNK target c-Jun impairs the expression of cluster of differentiation (CD)44, galanin or alpha7beta1 integrins, molecules known to be involved in regeneration [19].

To assess the correlation between JNK activity and axonal regeneration, we analysed GAP43-IR in DRGs after SNT. JNK expression and its activation persist for at least 30 days after axotomy if regeneration is blocked [10]; JNK activation in DRG neurons would thus initially promote survival after injury, and later would operate as part of a growth program [10]. In agreement with this, we document here that GAP43 immunoreactivity increases in axotomized primary sensory DRG neurons, and that this increase is partially prevented by D-JNKI-1 administration, or by deleting the genes for JNK1 and JNK3.

In primary somato-sensory neurons, CGRP is localized in peripheral axons, in their somata in the DRG, and in unmyelinated and thin myelinated central afferent fibers, where it has been implicated in the transmission/modulation of pain [43]. The role of GCRP, as well as that of substance P in neuropathic pain has been poorly investigated, partly because they are depleted after peripheral nerve lesion [23]. Impaired information-processing in the spinal cord may play an important role in the development of sensory abnormalities that result from peripheral nerve injury. It is likely that the lesion-induced downregulation of substance P and CGRP attenuates the transmission of peptide-related information at the first synapse in the dorsal horn; such changes may represent adaptive responses that limit the consequences of peripheral nerve damage to the organism as a whole, and that promote survival and recovery of individual neurons [44]. In animal models that investigate pathways that regulate inflammatory responses, a relationship between the JNK pathway and CGRP has been documented [22,23]. In agreement with previous studies [23], we report here that in axotomized primary sensory DRG neurons, CGRP activity is down-regulated. Moreover, our results following D-JNKI-1 treatment further underscore a relationship between JNK pathway activation and CGRP expression. We propose that, following nerve injury, activation of JNK pathway may influence CGRP regulation, mediated via JNK1 and JNK2 isoforms.

Studies on pain have primarily focused on neurons; however Zhuang et al. [9] demonstrated that spinal nerve ligation (SNL) also activates JNK1 in spinal astrocytes, and that such activation is essential for the maintenance of neuropathic pain. Glial changes, associated with an increased sprouting of primary nociceptive afferents (C and A-δ fibers) that enter the spinal cord, suggest a strict correlation between neuro-glial plasticity changes and peripheral sensitization [45,46]; these findings highlight the role of glia in pain transmission and suggests that JNK could also participate in this mechanism. In support, intratechal administration of TNF-α- activated astrocytes induces mechanical allodynia, which is preserved by D-JNKI-1 treatment [47]. Related to this, the relationship between NGF expression and JNK activation should be further investigated: on one hand, several reports claim that NGF, whose levels are increased in inflamed tissues, induces the activation of JNK in DRGs [24]. On the other hand, NGF administration prevents the facilitation of pain transmission at the level of the superficial laminae of the spinal cord [48].

### Role of JNK in pain behavior

Even though studies of knockout mice have some limitations, such as the occurrence of compensatory changes for the knocked out gene or the dependence of the phenotype on the background of mice strain [49], our findings exclude the possibility that the deletion of a specific JNK isoform results in abnormalities of locomotion or motor coordination, or in the alteration of the mechanical pain threshold. On the contrary, following SNT, KO mice displayed a decreased mechanical pain threshold compared to wild type animals: inhibition of JNK by D-JNKI-1 also had similar effects. However, JNK inhibition by D-JNKI-1 does not necessarily act as analgesic: since a JNK inhibitor can dramatically affect the production of cytokines [50], we suppose that it can function as an anti-hyperalgesic and/or as a neuromodulator, by restoring the baseline or normal pain threshold of the injured mice to which it is administered. Thus D-JNKI-1, instead of diminishing the stimulus-dependent response, would prevent that response, either by suppressing the pain-associated suffering or by directly reducing the responsiveness of nociceptors. D-JNKI-1 could thus be used to prevent and treat the onset of SNT-induced neuropathic pain.

D-JNKI-1 treated mice exhibit hypoalgesic responses to neuropathic pain within 30 days post- surgery, suggesting that JNK isoforms play a critical role in pain perception. These findings are consistent with recent results obtained using intrathecal or systemic delivery of D-JNKI-1 in another animal model for the study of neuropathic pain [9,27], in which the authors showed that JNK blockade prevents the development of neuropathic pain. Even though our work was aimed at investigating the role of JNK in modulating injury-induced cellular changes of DRGs in relation to concomitant pain behavior, we are aware of the involvement of supraspinal centers in the control and in the genesis of neuropathic pain. In fact, several reports refer changes in the expression of MAPKs, and namely of JNK, in somatosensory [51] and cingulate [52] cortex and in the hippocampus [53,54]. On the contrary, no changes were reported in the thalamus in a model of chronic pain in rats [55]. Although the parallel changes of DRG markers and pain behavior that we observed suggest that these phenomena may be causally related, in our experimental design, both in transgenic and in D-JNKI-1-treated mice single or all JNK isoforms were blocked in the entire brain. Therefore, further studies will be necessary to dissect the specific contribution of supraspinal centers in neuropathic pain and the relevant role played by JNK. Similarly, specific analysis and targeted manipulaitions of JNK expression or activity would be required to elucidate functional changes of intra- and supra spinal circuits involved in the pathogenesis of neuropathic pain.

### Autotomy behavior

Autotomy, or self mutilation can be interpreted either as a response to a persistent irritative stimulus, the failure to recognize the limb as self, or both. Our strain of mice, C57BL/6, displays a high autotomy score following sciatic nerve tight ligature [56]. Whereas JNK2 KO and wt mice displayed this behavior following SNT, mice with the JNK1 or JNK3 genes deleted, also mice treated with D-JNKI-1, displayed no autotomy lesions. This finding further supports a role for JNK in neuropathic pain. However, the increase in autotomy lesions which we observed in operated JNK2 KO mice apparently contradicts this view. Indeed, JNK2 KO mice show both GAP43 upregulation and the autotomy phenotype, whereas the other two strains failed to activate GAP43 and did not develop autotomy. Thus, GAP43 upregulation correlates with autotomy, which has been related to spontaneous pain [28]. This observation was somewhat surprising, since all JNK isoforms appear to be involved in the maintenance of neuropathic pain, as assessed by plantar aesthesiometer stimulation. However, it is likely that the appearance of autotomy and mechanical hyperalgesia following noxious plantar stimulation are elicited by different mechanisms that involve distinct JNK isoforms. In fact, autotomy score and Von Frey filament test (which is comparable to the automated Von Frey test we used in our study) reflect two different aspects of nociception: the first one measures a spontaneous behavior, whereas the second measures a cutaneous hypersensitivity to applied stimuli [57].

This is also consistent with our data on the prolonged effect of multiple D-JNKI-1 treatment on autotomy behavior, which does not occur with single injection, whereas both treatment protocols have the same results on mechanical hyperalgesia. Autotomy behavior might be elicited at higher levels as well: for example, it has been shown that autotomy behavior correlates with the expression of different dopamine receptors in the cingulate cortex [58].

## Conclusions

Our study confirms a role for JNK signaling in regulating pain threshold, and underscores that the deletion of all JNK isoforms can prevent the onset of neuropathic pain. Moreover, the deletion of a single splice JNK isoform is sufficient to mitigate established sensory abnormalities. Our data suggest that drugs, which selectively target individual *Jnk* gene products, can treat neuropathic pain that specifically results from axonal sprouting and pain transmission. Moreover, based on our behavioral observations, pharmacological JNK inhibition may offer a new option to prevent neuropathic pain associated with surgery that involves nerve damage.

## Methods

### Experimental animals

JNK1-, JNK2-, and JNK3-null mice, created by gene targeting [59-61] on the same C57BL/6, were kindly provided by Prof. Thomas Herdegen (Institute of Pharmacology, Kiel, Germany) and were bred in our animal facility. Homozygotes and heterozygotes were distinguished from C57BL/6 wild-type mice by using a polymerase chain reaction (PCR) assay. Animals were housed under a 12h light/dark cycle and provided food and water *ad libitum*. All experimental procedures on live animals were performed under the supervision of a licensed veterinarian, according to the European Communities Council Directive of 24 November 1986 (86/609/EEC) and University of Torino’s institutional guidelines on animal welfare (DL 116/92).

### Surgical technique and drug administration

Adult (2–4 months-old) male mice with a KO of the different JNK isoforms and age- matched wt mice, underwent sciatic nerve transection. They were administered isofluorane anesthesia plus O2 and N2O, the right sciatic nerve was exposed below the tendon of the obturator muscle. Without damaging the perineurium, the nerve was tightly ligated with 6–0 silk thread at two points separated by 4 mm and was transected between these points. The nerve was gently manipulated back into place and the incision closed with 4/0 suture.

To investigate whether JNK is involved in SNT-induced CGRP and GAP43 expression, the peptide JNK inhibitor D-JNKI-1 (0.3 mg/kg, 0.1 mM solution) was injected i.p. in a separate group of wt animals, 30 min before surgery. D-JNKI-1 was synthesized by linking the 10 amino acid human immunodeficiency virus Tat (48–57) transporter sequence to a 20 amino acid JNK-binding motif (JBD20) of JNK-interacting protein-1/islet-brain 1 (JIP-1). The peptide was also synthesized as protease-resistant D-form, to extend its half-life *in vivo*[13]. The peptide inhibitor is highly selective and does not affect the activity of 40 other protein kinases [13]. Based on previous reports of sciatic nerve lesions in mice [23], we decided that 72h is a suitable time interval for detecting axotomy-induced changes.

The number of mice/group was determined with a standard power analysis [62]. We set the significance level (probability of a type-I error) at 5% (α = 0.05) and sought to choose a sample size that would minimize the probability of type-II errors (β ≤ 10%). From our initial experiment, we obtained estimates of the expected effects of the 72h transection injury, and variability for a given number of mice. On the basis of this analysis, we determined that a sample size of four animals in each treatment group would be sufficient to detect a 30% difference in the immunoreactivities (described below) with a statistical power of 95%. In this paper, the injured and uninjured hindpaws are referred to as ipsilateral and contralateral hindpaws, respectively. Our controls were naive mice. All efforts were made to minimize the number of animals used and their suffering.

### Reverse transcription-PCR

Expression of Jnk1, Jnk2, Jnk3 and β-actin was analyzed by reverse transcription (RT)-PCR.Total RNA was extracted from DRGs with use of Trizol, according the instructions of the supplier (Invitrogen, Milan, Italy). Total RNA was treated with ribonuclease-free deoxyribonuclease I for 15 minutes at room temperature. The first- strand cDNA was synthesized with 5 μg of the total RNA and 500 ng of random primers using the Super Script first-strand synthesised system for reversetranscriptase–polymerase chain reaction (Invitrogen, Milan, Italy). The following primers were used: 5′-AAACAGGCCTAAATACGCTGGA-3′ and 5′-GACGGCTGCCCTCTTATGAC-3′ for Jnk1; 5′-GAGCTGGTGAAAGGTTGTGTGATATTCCA-3′ and 5′-AACAGTAATATACGGGTGGCGCAAG-3′ for JNK2; 5′-AACAATCGCTACACCTCCAAAGAC-3′ and 5′-TGGCAATAGATGACACATCCAGG-3′ for JNK3. β-actin mRNA expression was used as an internal standard, and the primers used for this were 5′- TCCATCATCATGAAGTGACGT-3′ and 5′-GAGCAATGATCTTGATCTTCAT-3′.

### Western blots

Animals were rapidly killed and L3-L6 DRGs were quickly removed and frozen in liquid nitrogen; they were mechanically homogenized in a hypotonic buffer containing a mixture of proteinase and phosphatise inhibitors (Sigma-Aldrich, St. Louis, MO). Protein sample (30 μg) were separated on 12% SDS gel and transferred onto PVDF membrane. The blots were blocked with 5% BSA and incubated overnight at 4°C with the following antibodies i) a polyclonal rabbit anti pJNK (1:500, Cell Signaling Technology, Beverly, MA, Cat No. #9251, lot.10). This antibody was produced by immunizing rabbits with a synthetic phospho-peptide corresponding to residues surrounding Thr183/Tyr185 of human JNK. pJNK was purified by protein A and peptide affinity chromatography. It detects endogenous levels of p46 and p54 JNK dually phosphorylated at Thr183 and Tyr 185 [9]. ii) a polyclonal rabbit anti total JNK (1:500,Cell Signaling Technology, Beverly, MA, Cat No. #9252, lot.8). This antibody was produced by immunizing animals with a GST/human JNK2 fusion protein. The antibodyis purified by protein A and peptide affinity chromatography, and detects endogenous levels of p46 and p54 JNK [9]. Following incubation in primary antibody, blots were further incubated with stabilized HRP-conjugated goat anti-rabbit IgG (H + L) secondary antibody (1:1000, Pierce, Rockford, USA, Cat No. 32460), and HRP activity was then detected with Super Signal West Dura Extended Duration Substrate (Pierce, Rockford, USA). For loading controls, the blots were probed with monoclonal anti-acetylated tubulin antibody Clone 6-11B-1 (1:10.000, Sigma, Missouri, USA, Cat No. T6793).

### Immunohistochemistry

After appropriate post-surgery survival times, animals were killed with an overdose of anaesthetic (3% chloral hydrate in saline) and perfused transcardially with 4% paraformaldehyde in 0.1 M phosphate buffer (PB, pH 7.4). L4 DRGs were removed and postfixed in the same fixative for two hours. The tissue was cryoprotected by immersion over night in buffered 30% sucrose, embedded, and frozen on cryostat medium (Bio-Optica, Milan, Italy). DRG sections (10 μm) were serially cut in a cryostat, mounted on TESPA (3-aminopropyl-trithoxysilan; Fluka, St. Louis, MO, USA) coated slides and processed for immunofluorescence staining.

Sections were incubated at 4°C overnight with i) a polyclonal rabbit anti GAP43 antibody (Abcam, Cambridge, UK, Cat No. ab7462, lot.716099) diluted 1:250 in 1,5% NDS PBS 0.3% Triton X-100. The antibody was obtained by immunizing a rabbit with the Keyhole Limpet haemocyanin-coupled synthetic peptide C- KEDPEADQEHA, corresponding to amino acids 216–226 of rat GAP43. On conventional Western blot analysis of cultured cortical neurons, this antibody visualizes a single band at 43 kDa [63]. ii) A polyclonal rabbit antiserum to Calcitonin Gene-Related Peptide (CGRP; Biomol, Plymouth, PA, USA, Cat No. CA1134, lot. Z05177), diluted 1:500 in phosphate-buffered saline (PBS) containing 5% normal donkey serum (NDS; Sigma) and PBS 0.3% Triton X-100. This antibody detects peptidergic nociceptive neurons, and was partially purified by caprylic acid and ammonium sulphate precipitation. Previous studies [64] show that CGRP (CA1134) produces a similar pattern of immunoreactivity in lumbar DRGs after SNT.

After rinsing, primary antibodies were detected by incubating sections for one hour at room temperature in 1:200 Cy3-coniugated donkey anti-rabbit IgG (H + L) (Jackson Immuno Research Laboratories, West Grove, PA, USA) or in 1:100 Cy2-coniugated donkey anti-rabbit IgG (H + L) (Jackson Immuno Research Laboratories). Sections were counterstained by incubation with DAPI (4,6-Diamidino-2-phenyindole, dilactate; Sigma) diluted 1:10 in PBS 0.3% Triton X-100 for 10 minutes at room temperature after immunohistochemical labelling. Controls included sections treated with secondary antibody alone, which did not show appreciable staining.

### Quantitative morphometric analyses

Computer-assisted quantitative analyses of the CGRP and GAP43 immunoreactivities was assessed at 20x with a Nixon Eclipse 600 light microscope coupled with a computer-assisted image analysis system (Neurolucida software, Micro Bright Field, Williston, VT, USA). The total number of positive neuron profiles was divided by the total number of DAPI stained profiles in the L4 DRG sections, and the percentage of immunoreactive neurons was calculated. Percentages obtained from 8–10 sections from each animal were averaged (at least three animals for each neurochemical marker). Every section was digitized, and images were acquired via red, green and blue channels, with a 512x512 eight-bit matrix. Each pixel was assigned a luminance value ranging from 0 to 255 in each channel. The lower threshold value was determined from the unstained portions of the immunoreacted sections and from control sections,the upper thresholdvalue was established from immunopositive cells that highly express fluorochromeactivity. Only neurons with visible nuclei and a brightness value of at least 20 (after subtraction of background staining) were included in counts.

### Behavioral testing

Animals were habituated to the testing environment daily for one week before baseline testing. In all studies, the observer was blind to the genotype of the mice, and to the how each animal had been treated.

### Walking tracks analysis

We used locomotor activity as an index of normal behavior. Mice were evaluated to obtain three footprint parameters i) toe spread, or the distance between the first and fifth toes; ii) print length, or the distance between the tip of the toe and the most posterior part of the foot that was in contact with the ground; iii) stride length. The assessments were carried out in two ways. Animals were first placed on plexiglass corridor (60cm x 4.5 cm); a video camera was placed under the transparent corridor to record the walking tracks of each mouse. The animal’s cage was placed at the end of the corridor to induce the animal to walk to it. The hind feet of the mice were dipped in ink and the animals were allowed to walk along the corridor, which was lined with ordinary white paper. For each animal, a mean of three footprints was used in the assessment.

### Rotarod test

Locomotor activity was also tested using arotarod apparatus (Model 760; UgoBasile, Comerio, Italy): mouse’s ability to stay on a rotating rod that accelerated initially from 4 rpm to a final speed of 32 rpm was assessed; the cut-off time for the test was 300 sec. Mice were pretrained in the same rotarod apparatus at a constant speed of 15 rpm once a day for 7 days before baseline testing.

### Open field test

The open field test was performed in a cube (50x50x50 cm) made of gray polyacrylic plastic plates; it had no ceiling. Each mouse was transferred from its cage to a corner of the arena, and its activity was recorded with a video camera. The arena was cleaned with 70% ethanol and water between trials. Horizontal activities were assessed for 10 min, and analyzed using the EthoVision video tracking system (Noldus). The center point of the body of a freely moving mouse was captured to obtain the total distance travelled in the arena, and quantitatively describe the animal’s open field exploratory behavior.

### Neuropathic pain test

Hyperalgesia induced by SNT was measured with use of an automatic von Frey apparatus (Dynamic Plantar Aesthesiometer, Model 37450, UgoBasile). On each testing day, the nociceptive threshold of the hindpaws, expressed as the force (in grams) at which the mouse withdrew its paw in response to the mechanical stimulus, was recorded. For habituation, mice were placed in plastic cages with a wire net floor, 5 min before the experiment. The mechanical stimulus was applied to the mid-plantar surface of the hindpaw to induce a slight pressure to the skin, as described [65,66]. Ipsilateral and contralateral withdrawal thresholds were taken as the mean of three consecutive measurements per paw, with a 10 s interval between each measurement.

A cut-off force of 20 grams was used, one we had ascertained that no injury was produced within this force range. Mice were tested from day 2 pre-injury to day 30 post-injury.

### Autotomy score

Autotomy was assessed at all time-points after surgery, with use of the scale described by Wiesenfeld and Hallin [67]. Scoring points ranged from 0 to 5, where 0 = normal undamaged hindpaw; 1 = the tips of one or more nails were removed; 2 = one or more nails totally removed and damage to the distal portion of one or more digits; 3 = one or more digits totally removed; and 4 = one or more digits totally removed plus damage to the foot. No animals scored higher than 4.

### Statistical analysis

Statistical analyses were performed using GraphPad Prism version 4.0 (GraphPad software, San Diego, CA). All values were presented as mean + standard error of the mean (SEM). Means were compared by one way analysis of variance (ANOVA) and Student’s t-test with Bonferroni as a *post-hoc* test. Behavioral data related to hyperalgesia were analyzed using two-way ANOVA of repeated measures, followed by a Tukey–Kramer *post-hoc* comparison. Differences were considered significant at p < 0.05 level.

## Abbreviations

PK, Protein Kinase; MAPKs, Mitogen-Activated Protein Kinases; ERK, Extracellular Signal-Regulated Kinase; JNK, c-Jun N-terminal Kinase; DRGs, Dorsal Root Ganglia; D-JNKI-1, D-form of the JNK binding domain of JNK-Interacting protein-1; STF, Activating Transcription Factor; Elk-1, EtsLiKe gene-1; SNT, Sciatic Nerve Transection; AP-1, Activator protein 1; GAP-43, Growth Associated Protein; KO, Calcitonin Gene Related Peptide; NGF, Neuronal Growth Factor; dpo, Days Post Operation; KO, KnockOut; wt, wild-type; RT-PCR, Reverse Transcription; p, phosphorylated; Thr, Threonine; Tyr, Tyrosone; IR, ImmunoReactive; SNL, Spinal Nerve Ligation; MAP, Microtubules Associated Protein; CD, cluster of differentiation.

## Competing interests

The authors declare that they have no competing interests.

## Authors’ contributions

GM designed, performed the experiments and wrote the manuscript together with AV. IR performed part of the experiments. SC contributed to behavioral tests and statistical analysis. VV contributed to the RT-PCR. CB contributed to design of experimentation and of drug administration. AV and FR supervised the experiments. All authors read and approved the final manuscript.

## Supplementary Material

Additional file 1 **Walking track analysis of JNK KO mice.** Locomotor activity of JNK KOs was similar to the wt mice as measured by comparing the stride length of all tested animals. Data are presented as mean + SEM.p = 0.92 by one-way ANOVA; n = 48. Click here for file

Additional file 2 **Open field behavior of JNK KO mice.** Mice were placed in an open field box and movements and behaviors were recorded for 10 min using a video camera in the vertical plane. There were no differences between JNK KO and wt mice in the total distance travelled in the arena. Data are presented as mean + SEM. P = 0.91 by one-way ANOVA; n = 48. Click here for file
