# Supplementary figures and images for: Role of JNK isoforms in the development of neuropathic pain following sciatic nerve transection in the mouse
Source: Mol Pain. 2012 May 22;8:39. doi: 10.1186/1744-8069-8-39 (PMC3436729; doi:10.1186/1744-8069-8-39)

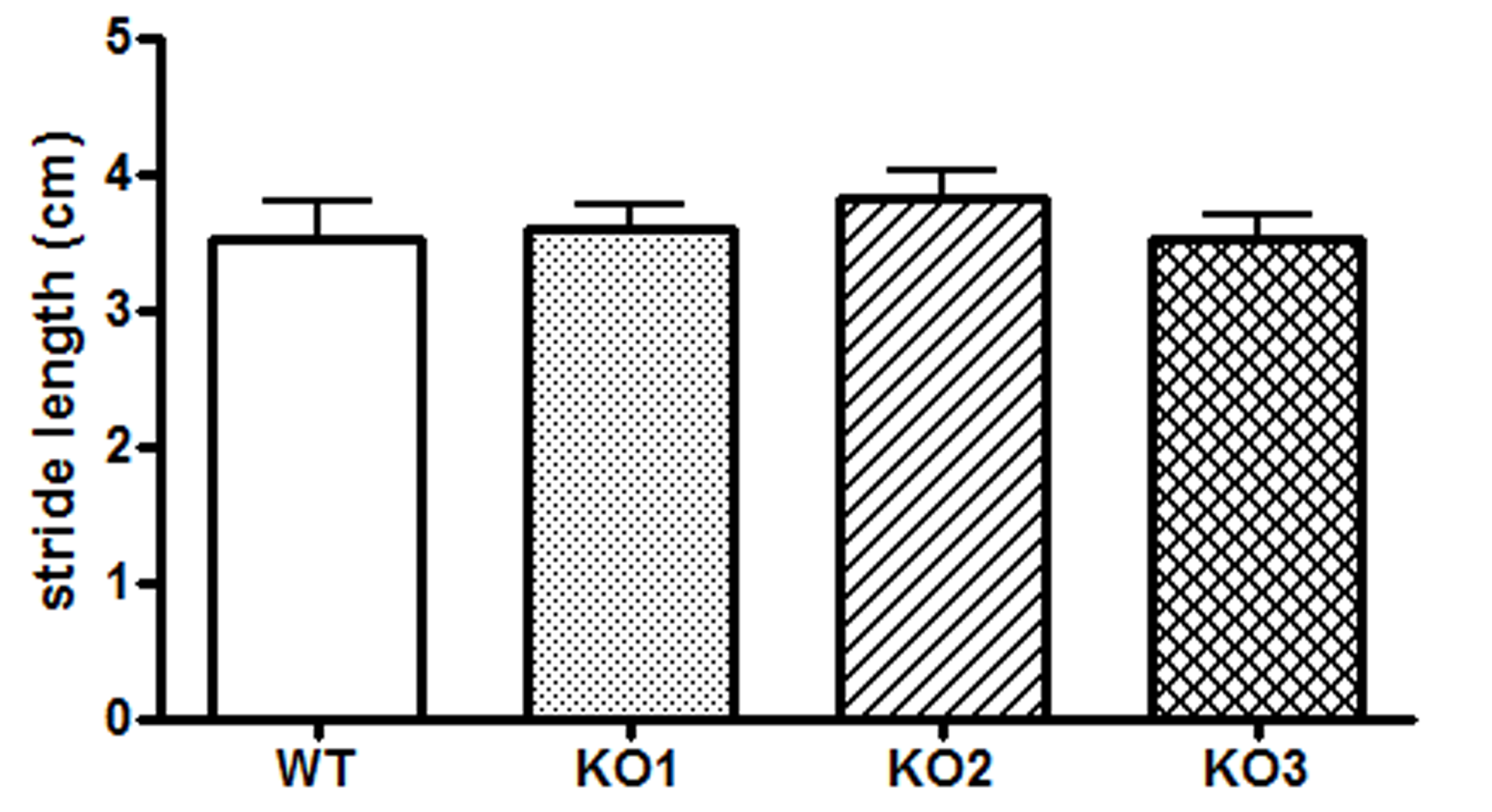

Supplement: Additional file 1 — Walking track analysis of JNK KO mice. Locomotor activity of JNK KOs was similar to the wt mice as measured by comparing the stride length of all tested animals. Data are presented as mean + SEM.p = 0.92 by one-way ANOVA; n = 48. [file 1744-8069-8-39-S1.tiff]

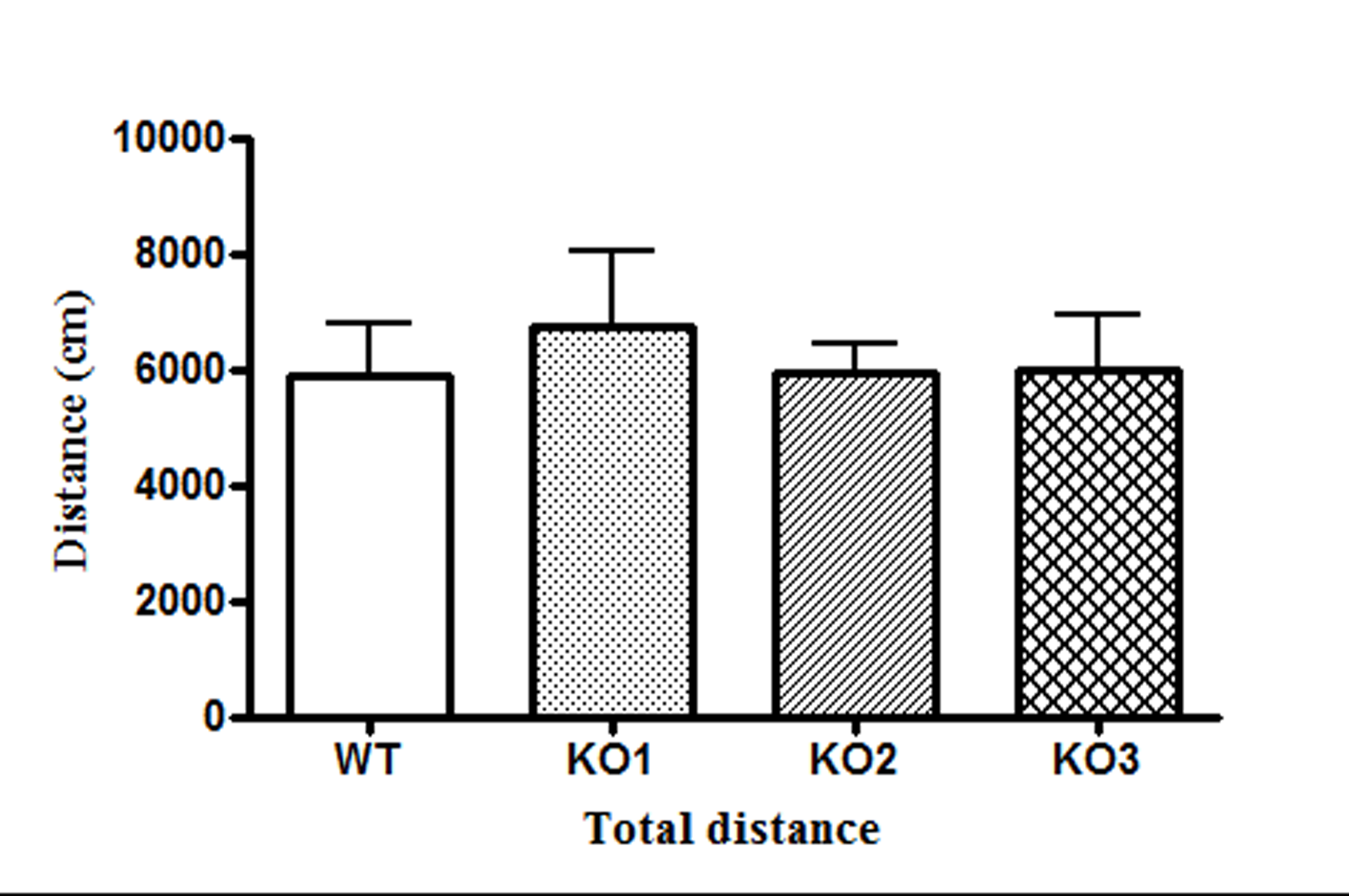

Supplement: Additional file 2 — Open field behavior of JNK KO mice. Mice were placed in an open field box and movements and behaviors were recorded for 10 min using a video camera in the vertical plane. There were no differences between JNK KO and wt mice in the total distance travelled in the arena. Data are presented as mean + SEM. P = 0.91 by one-way ANOVA; n = 48. [file 1744-8069-8-39-S2.tiff]
